# Essential Amino Acid Enriched High-Protein Enteral Nutrition Modulates Insulin-Like Growth Factor-1 System Function in a Rat Model of Trauma-Hemorrhagic Shock

**DOI:** 10.1371/journal.pone.0077823

**Published:** 2013-10-28

**Authors:** Xianfeng Xia, Xinying Wang, Qiurong Li, Ning Li, Jieshou Li

**Affiliations:** Research Institute of General Surgery, Jinling Hospital, Medical School of Nanjing University, Nanjing, Jiangsu Province, China; University College London, United Kingdom

## Abstract

**Background:**

Nutrition support for critically ill patients supplemented with additional modular protein may promote skeletal muscle protein anabolism in addition to counteracting acute nitrogen loss. The present study was designed to investigate whether the essential amino acid (EAA) enriched high-protein enteral nutrition (EN) modulates the insulin-like growth factor-1 (IGF-1) system and activates the mammalian target of rapamycin (mTOR) anabolic signaling pathway in a trauma-hemorrhagic shock (T-HS) rat model.

**Methodology/Principal Findings:**

Male Sprague-Dawley rats (n = 90, 278.18±0.94 g) were randomly assigned to 5 groups: (1) normal control, (2) pair-fed, (3) T-HS, (4) T-HS and standard EN, and (5) T-HS and EAA enriched high-protein EN. Six animals from each group were harvested on days 2, 4, and 6 for serum, gastrocnemius, soleus, and extensor digitorum longus sample collection. T-HS significantly reduced muscle mass. Nutrition support maintained muscle mass, especially the EAA enriched high-protein EN. Meanwhile, a pronounced derangement in IGF-1-IGFBPs axis as well as impaired mTOR transduction was observed in the T-HS group. Compared with animals receiving standard EN, those receiving EAA enriched high-protein EN presented 18% higher serum free IGF-1 levels following 3 days of nutrition support and 22% higher after 5 days. These changes were consistent with the concomitant elevation in serum insulin and reduction in corticosterone levels. In addition, phosphorylations of downstream anabolic signaling effectors - including protein kinase B, mTOR, and ribosomal protein S6 kinase1 - increased significantly in rats receiving EAA enriched high-protein EN.

**Conclusion/Significance:**

Our findings firstly demonstrate the beneficial effect of EAA enriched high-protein EN on the metabolic modulation of skeletal muscle protein anabolism by regulating the IGF-1 system and downstream anabolic signaling transduction.

## Introduction

Increased nitrogen loss and skeletal muscle wasting are metabolic features in patients under catabolic stress. Nearly 20% of total body protein can be lost in critically ill patients [1], and this severely negative nitrogen balance can adversely affect morbidity and mortality. The erosion of lean body mass is undoubtedly multifactorial [2], [3]; but it is in part attributed to the reduction in circulating insulin-like growth factor-1 (IGF-1) levels or IGF-1 signaling [4], [5]. In normal physiological state, IGF-1 bonds to the type-1 cell-surface receptor and then exerts an anabolic effect by activating the phosphatidylinositol 3-kinase (PI3K)-protein kinase B (PKB/Akt)-mammalian target of rapamycin (mTOR) pathway [6], [7]. Stimulation of mTOR complex1 (mTORC1) contributes to the phosphorylations of two key downstream effectors, eukaryotic initiation factor 4E-binding protein 1 (4E-BP1) and p70 ribosomal protein S6 kinase1 (p70S6K1), which then induce protein translation initiation [8], [9]. The sustained decrease in circulating IGF-1 levels under catabolic states is thus believed to be responsible, at least in part, for the impaired IGF-1 bioactivity and damaged muscle protein anabolism.

IGF-1 circulating in the peripheral vasculature bonds to specific binding proteins. Approximate 95% of circulating IGF-1 forms a ternary complex consisting with insulin-like growth factor binding protein 3 (IGFBP-3) and an acid-labile subunit (ALS) [10]. This ternary complex regulates the biologic effect of IGF-1, functioning as a storage reservoir of IGF-1 in plasma and extending its half-life.

Endogenous IGF-1 synthesis is quite sensitive to nutrient availability. In previous work, a 60% reduction in plasma IGF-1 concentrations as well as 8% decrease in gastrocnemius mass was observed in rats receiving a protein-free diet, while recovering to normal after standard casein diet refeeding [11]. Dietary protein intake is also associated with IGF-1 status and whole-body nitrogen economy [12]. In patients with recent hip fracture, a significant elevation of serum IGF-1 levels was observed as early as 7 days after 20 g/(kg·d) protein supplementation [13]. Meanwhile, clinical study revealed that amino acid infusion during anesthesia could attenuate the decrease in IGF-1 levels and maintain glucose homeostasis after surgery [14]. These results collectively indicated that elevated serum IGF-1 levels appeared to be associated with the response to protein or amino acid supplementation during fasting or stress period. In addition, Takenaka *et al.* [15] reported that dietary restriction of signal essential amino acid (EAA) decreased IGF-1 production, suggesting that adequate EAA in diet might be required for the IGF-1 synthesis. A recent study regarding acute regulation of the IGF-1 system by macronutrients further demonstrated that EAA/carbohydrate mixture ingestion following high-intensity exercise promoted a significant increase in circulating free IGF-1 levels [16].

The essential role of nutrition support for critically ill patients is to promote protein anabolism and protect the lean body mass and function. Historically, providing equivalent amounts of protein or amino acids in nutrition support attempted to counteract the dramatic nitrogen loss [17]. However, it remains to be elucidated whether adequate protein intake modulates the stress response and muscle protein anabolism during critical illness through the IGF-1 system regulation.

Therefore, the purpose of the present study was to investigate the role of long-term enteral nutrition (EN), in particular EAA enriched high-protein EN, upon the regulation of IGF-1 system function and downstream mTOR-related signaling transduction in a Sprague-Dawley (SD) rat model of trauma-hemorrhagic shock (T-HS). Serum levels of insulin and corticosterone were also measured, since both factors were implicated in the regulation of IGF-1 by their respective roles in IGFBP regulation [18].

## Materials and Methods

### Animals

Male SD rats weighted 278.18±0.94 g (Medical Experiment Animal Center of Jinling Hospital, Nanjing, China) were housed in a temperature (25∼28°C) and light (12∶12-hour light-dark cycle) controlled environment for 1 week prior to the experiment. Water and standard chow diet were provided *ad libitum*. All procedures were carried out in accordance with the “Guide for the Care and Use of Laboratory Animals” published by the National Institutes of Health (NIH publication 86-23 revised 1985) and following protocols approved by the Animal Care and Use Committee of Jinling Hospital.

### Surgical Procedure and Experimental Design (Figure 1)

Following the acclimatization period, rats were fasted overnight (Day-1). All surgeries were performed using sterile technique. At day 0, rats were anesthetized by intraperitoneal injection of ketamine (100 mg/kg body weight (BW)) and placed on a temperature-controlled heating pad. A midline laparotomy (5 cm) was made to insert a catheter (ID 0.8 mm, OD 1.2 mm) into the anterior wall of the stomach followed by suturing to the stomach wall and exteriorizing through the anterolateral abdominal wall. The catheter was then subcutaneously tunneled to the shoulder region, and connected through a customized rotating swivel to a 50 mL syringe pump (Research Center for Analytical Instrument, Zhejiang University, China). An additional groin incision was made. The left femoral artery was catheterized for bleeding, monitoring mean arterial pressure (MAP), and fluid resuscitation through a three-way microvalve. Rats were bled to a target MAP of 30∼35 mmHg within 15 minutes and maintained for 45 minutes. Blood pressure was recorded in 5-minute intervals. At the end of hemorrhagic shock period, rats were resuscitated by infusing their shed blood and lactated Ringer’s solution in a 1∶2 ratio. All rats were allowed food and water *ad libitum* overnight.

**Figure 1:**
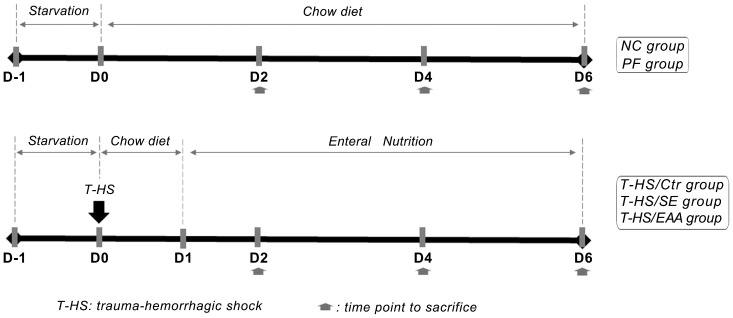
Experimental design. This study includes five groups. After undergoing the trauma-hemorrhagic shock (T-HS) operation, rats received either standard enteral nutrition (EN) (T-HS/SE), essential amino acid (EAA) enriched high-protein EN (T-HS/EAA), or a continue infusion of isotonic saline but on normal chow diet (T-HS/Ctr). Food intake in a pair-fed group (PF) was restricted to the T-HS/Ctr group. A normal control group (NC) without T-HS and fed *ad libitum* chow was also included. Six individuals in each group were harvested on days 2, 4, and 6, respectively.

Animals were randomized to 5 groups. At T-HS 3 groups, rats received either standard EN (T-HS/SE, n = 18), EAA enriched high-protein EN (T-HS/EAA, n = 18), or a continue infusion of isotonic saline but on normal chow diet (T-HS/Ctr, n = 18). One group that was pair-fed (PF, n = 18) with the T-HS/Ctr group was set to control for the influence of reduced food intake post-operatively. Rats in the normal control group (NC, n = 18) were under no treatment and fed standard chow diet *ad libitum* throughout the study.

### Nutrition Program

Following overnight recovery (Day 1), chow diet was removed from rats in the T-HS/SE and T-HS/EAA groups. A continuous EN infusion was started and provided for the subsequent 5 days, providing a non-protein calorie of 250 kcal/(kg BW·d). The T-HS/SE rats received ENSURE® (Lot# 10127NR, Abbott Laboratories B.v., Zwolle, Netherlands) (64.38 g/(kg BW·d), providing 1.65 g N/(kg BW·d)) as the standard enteral formula, in which the ratio of non-protein calorie to nitrogen is 152∶1 **(** **Table 1** **)**. Additional nitrogen supplementation in the T-HS/EAA group was provided by leucine enriched EAA solution containing 0.849 g N/(kg BW·d) based on the ENSURE® diet. Therefore, the ratio of non-protein calorie to nitrogen decreases to 100∶1. The composition of the EAA mixture is provided in **Table 2**, with minor modifications to previous studies [19], [20]. Half of the estimated requirement based upon individual BW was administrated on the first day and the full requirement was provided subsequently.

**Table 1:** Nutrition composition of standard diet: ENSURE®.

Nutrition Composition	/100 g formula
Energy, *kcal*	450
Protein, *g*	15.9
Fat, *g*	15.9
Linoleic acid, *g*	8.7
Carbohydrate, *g*	60.7
Moisture, *g*	5

**Table 2:** The composition of the essential amino acid mixture.

Essential amino acids	Percentage (%)
Lysine	13%
Tryptophan	8%
Phenylalanine	8%
Leucine	38%
Isoleucine	9%
Valine	11%
Threonine	10%
Methionine	3%

In the T-HS/Ctr group, rats received saline at a constant rate of 0.3 mL/(100 g BW·h) through the enteral routine and had free access to chow diet. Food intake in PF rats was restricted to the T-HS/Ctr group. All rats were allowed free access to water.

BW and the amount of EN solution infused were recorded daily. Frequency of diarrhea was also observed to evaluate the tolerance of EN in the T-HS/SE and T-HS/EAA rats.

### Sample Collection

Rats (n = 6) were euthanized from each group on days 2, 4, and 6, respectively. EN was stopped 1 hour before sacrifice in the T-HS/SE and T-HS/EAA groups. After anesthesia, blood was collected by cardiac puncture. Serum was isolated by centrifuge at 4000 rpm at 4°C for 10 minutes and then kept at −80°C until further measurements.

The gastrocnemius, soleus, and extensor digitorum longus (EDL) of the right leg were dissected out, weighted, wrapped, and then snap frozen in liquid nitrogen within 3 minutes. All samples were then stored at −80°C until analysis.

### Determination of Serum Free IGF-1, IGFBP-1, IGFBP-3, Insulin, and Corticosterone Levels by Enzyme-linked Immunosorbent Assay (ELISA)

In contrast to measuring total serum IGF-1 levels, we utilized a commercial IGF-1 ELISA kit from R&D Inc. (Minneapolis, MN, USA) to determine the free serum IGF-1 concentrations, which represent the “bioavailable” portion of the circulating IGF-1 pool [16]. Serum concentrations of IGFBP-1, IGFBP-3, insulin, and corticosterone were also determined by ELISA kits (R&D Systems, Minneapolis, MN, USA) in accordance with the manufacturer’s instructions.

### Western Blot

Muscle preparation and western blotting analysis were performed as previously detailed [21]. Briefly, ∼ 80 mg of frozen muscle sample was homogenized in RIPA lysis buffer (25 mM Tris-HCl (pH 7.6), 150 mM NaCl, 1% NP-40, 1% sodium deoxycholate, 0.1% SDS) containing 200 mM NaF, 1 mM Na_3_VO_4_, 25 mM β-glycerophosphate, 1 mM PMSF, and 1% protein inhibitor Cocktail (Sigma-Aldrich Inc., St. Louis, MO, USA ) for 30 minutes on ice. The lysate was then centrifuged at 12,000 g at 4°C for 8 minutes, and the supernatant was collected. Total protein concentration was determined using the BCA assay (Sangon Biotech Co., Shanghai, China). The supernatant was diluted (4∶1) in a 5× loading buffer (Beyotime Institute of Biotechnology Co., Nantong, China) and then boiled at 100°C for 5 minutes.

Equal amounts of total protein (75 ug) were separated by 8% or 10% sodium dodecyl sulfate-polyacrylamide gel electrophoresis (SDS-PAGE) determined by the size of target protein and then transferred to polyvinylidene difluoride membrane (Millipore Co., Billerica, MA, USA). Blots were blocked in 5% BSA for 1 hour at room temperature and incubated in specific primary antibodies overnight at 4°C. Blots were then washed in TBST (Tris-buffered saline and 0.05% Tween-20) three times followed by incubation with secondary antibody for 2 hours at room temperature. The protein bands were visualized by a chemiluminescence detection system (Pierce Biotechnology Inc., Rockford, IL, USA) with exposure to Kodak XAR film (Eastman Kodak). Band density was analyzed using Quantity One 4.6.2 software (BioRad). Specific total protein was re-probed after stripping the phospho-primary and secondary antibody. Phosphorylation data are described relative to the total protein expression after normalization by internal loading control.

### Antibodies

Antibodies that detected α-tublin (1∶1000), phospho-mTOR (Ser2448; 1∶500), S6K1 (1∶1000), phospho-S6K1 (Thr389; 1∶500), and phospho-Akt (Ser473; 1∶1000) were from Cell Signaling Technology (Beverly, MA, USA). Akt (1∶1000) and mTOR (1∶1000) antibodies were from Abcam (Cambridge, UK). Anti-rabbit IgG horseradish peroxidase-conjugated secondary antibody (1∶3000) was from Kirkegaard & Perry Laboratories (KPL) Biotechnology Inc. (Gaithersburg, ML, USA).

### Determination of Serum Amino Acid Concentrations by High-performance Liquid Chromatography (HPLC)

Serum amino acid concentrations were assayed by a Waters Aliance 2695 HPLC system (Waters, MA, USA) after pre-column derivatization with phenylisothicyanate (PITC) (Thermo Scientific (Pierce), Rockford, IL, USA) by our previous method [22]. Data were calculated on the basis of external amino acid standard (Sigma-Aldrich Inc., St. Louis, MO, USA).

### Statistical Analysis

Results are expressed as mean ± SE. Repeated-measures ANOVA was used to characterize dietary intake and BW gain during the 6-day recovery period. For other purposes, statistical evaluation among groups was performed by one-way ANOVA followed by LSD post-hoc analysis, using SPSS software package (SPPS Inc., Chicago, IL, USA). In the case of heterogeneous variances, we used Dunnet’s T3 test. A *p* value <0.05 was considered significant.

## Results

### 1. Growth Assessment during Study

**Figure 2:**
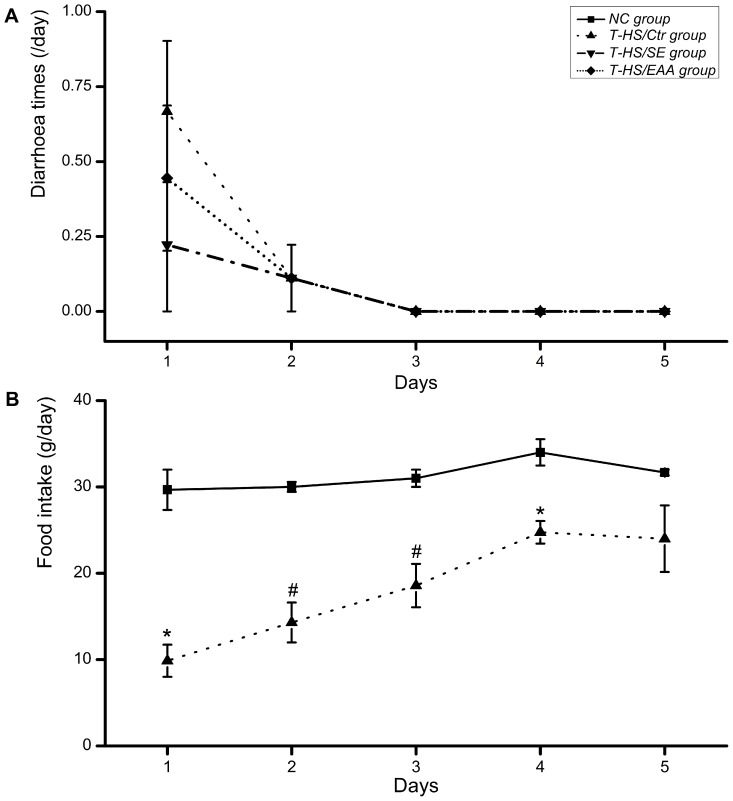
Observations of diarrhea and food intake in normal or T-HS injured animals. Diarrhea times (A) and food intake (B) in specific groups were recorded daily during study period. Values are presented as mean ± SE. Marker indicates a significant difference from NC group. *, *p*<0.05; ^#^, *p*<0.01. T-HS, trauma-hemorrhagic shock.

The average incidence of diarrhea was 0.44/day on the first day following T-HS, and gradually disappeared at day 3 **(** **Figure 2A** **)**. Early EN failed to increase the incidence of diarrhea, indicating that rats in the T-HS/SE and T-HS/EAA groups tolerated the nutrition support. After T-HS, rats consumed less chow (*p*<0.05) for 4 days but achieved normal by day 5, when the food intake did not differ between the T-HS/Ctr and NC groups **(** **Figure 2B** **)**. The daily caloric intake of the T-HS/SE and T-HS/EAA groups is additionally provided in **Table 3**.

**Table 3:** Daily caloric intake in enterally fed rats (kcal/day).

Groups	Time point				
	Day 1	Day 2	Day 3	Day 4	Day 5
T-HS/SE	33.03±0.18	65.21±0.98	67.79±0.66	69.25±0.98	69.69±0.87
T-HS/EAA	32.67±0.17	65.75±0.25	68.42±0.17	69.25±0.25	70.67±0.60

**Figure 3:**
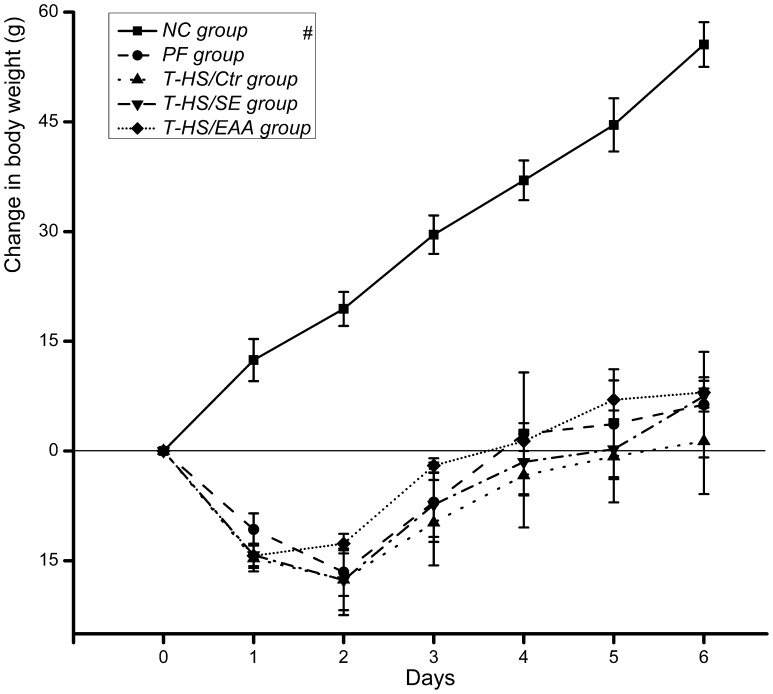
Changes in body weight of the animals. Values are presented as mean ± SE. Marker indicates a significant difference from other groups across study period. ^#^, *p*<0.01.

Animals weighed an average of 278.18±0.94 g at the beginning of the study. BW was low in all T-HS injured rats throughout the study, but did increase with time **(** *p*<0.01, **Figure 3** **)**. Rats in the T-HS/Ctr group presented a relatively slow gain of BW during the recovery period. Nutrition support was thought to be beneficial for achieving weight gain, especially the EAA enriched high-protein EN, however no significant difference between groups was observed at any time point.

**Figure 4:**
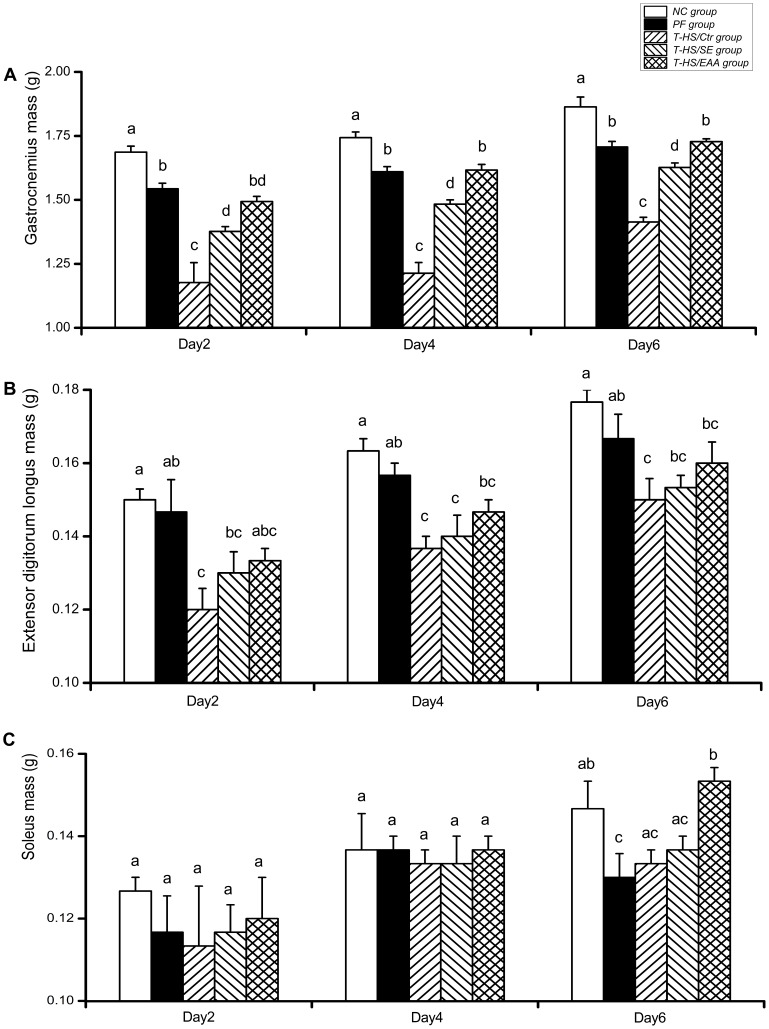
Comparison of skeletal muscle mass. Wet masses of gastrocnemius (A), extensor digitorum longus (B), and soleus (C) in each group were measured following 2, 4, and 6 days of recovery from T-HS. Values are presented as mean ± SE. Groups with different letters at each time point indicate a significant difference (*p*<0.05). T-HS, trauma-hemorrhagic shock.

T-HS induced significant reductions in gastrocnemius and EDL masses **(** **Figure 4A, B** **)**. Gastrocnemius masses in the T-HS/SE and T-HS/EAA groups were significantly higher than the T-HS/Ctr group by 2 days post-T-HS (*p*<0.01), and this trend continued throughout the remainder of the study. In addition, the T-HS/EAA group exhibited a significant increase in gastrocnemius mass compared with the T-HS/SE group on days 4 and 6, respectively (*p*<0.01). However, both EN treatments had limited improvement in EDL mass maintenance. For soleus mass, a slight but without significant decrease was observed after T-HS **(** **Figure 4C** **)**. EAA enriched high-protein EN caused a significant increase in soleus wet muscle mass by day 6, which was comparable with the NC group (*p*<0.05).

### 2. Serum Free IGF-1, IGFBP-3, and IGFBP-1 Concentrations

**Figure 5:**
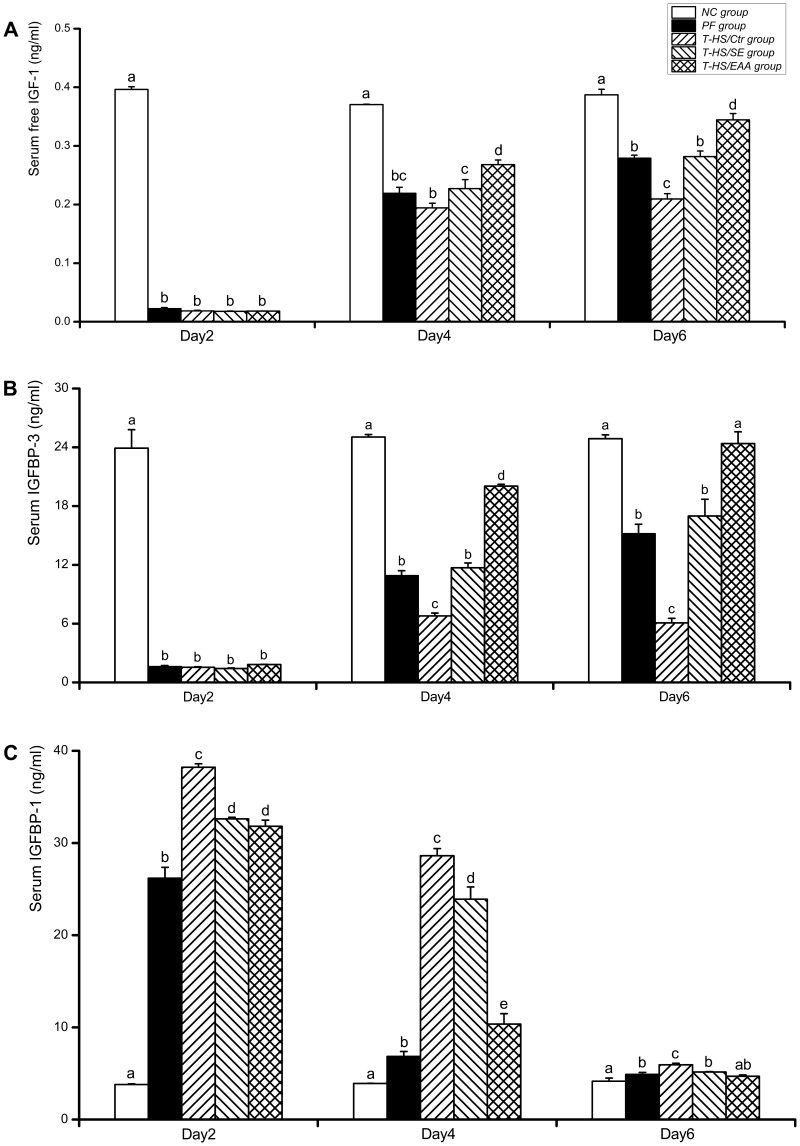
ELISA analysis of IGF-1-IGFBPs axis expression. Serum concentrations of free IGF-1 (A), IGFBP-3 (B), and IGFBP-1 (C) in each group were measured following 2, 4, and 6 days of recovery from T-HS. Values are presented as mean ± SE. Groups with different letters at each time point indicate a significant difference (*p*<0.05). T-HS, trauma-hemorrhagic shock.

Serum free IGF-1 concentrations in the PF, T-HS/Ctr, T-HS/SE, and T-HS/EAA groups were significantly decreased by day 2 **(** *p*<0.01, **Figure 5A** **)**. Following this time point, free IGF-1 concentrations increased gradually, but most slowly in the T-HS/Ctr group, which remained statistically lower than the other EN-treated groups (vs T-HS/SE at day 4, *p*<0.05; vs T-HS/EAA at day 4, *p*<0.01; vs T-HS/SE or T-HS/EAA at day 6, *p*<0.01). In addition, lower free IGF-1 levels were measured in the T-HS/Ctr group than PF group at day 4 and it reached statistical difference by day 6 (*p*<0.01), indicating that the depressed IGF-1 expression was sustained 6 days following T-HS after eliminating the interference of reduced food intake. Furthermore, free IGF-1 levels were 18% higher in the T-HS/EAA group than T-HS/SE group following 3 days of nutrition support (*p*<0.05). And a greater magnitude was observed by day 6 (122%, vs T-HS/SE; *p*<0.01).

The pattern of serum IGFBP-3 concentrations between groups was similar to that observed in free IGF-1 **(** **Figure 5B** **)**. In the T-HS/Ctr group, IGFBP-3 levels remained lower than the PF group on days 4 and 6, respectively (*p*<0.01). Following enteral feeding, there was a significant elevation in IGFBP-3 levels compared with the T-HS/Ctr group (*p*<0.01). Moreover, EAA enriched high-protein EN-fed rats exhibited a 170% and 140% increase in IGFBP-3 levels compared with animals receiving standard EN on days 4 and 6, respectively (*p*<0.01).

Serum IGFBP-1 levels in the NC group were markedly lower than the other groups at each time point, with the exception of day 6 when IGFBP-1 levels in the T-HS/EAA group decreased to levels comparable with the NC group **(** **Figure 5C** **)**. T-HS increased IGFBP-1 concentrations in the T-HS/Ctr group by 46% compared with the PF group at day 2 (*p*<0.01), and this trend was likely to continue throughout the study period. Although both groups of enterally fed rats exhibited significant decreases in IGFBP-1 levels compared with rats in the T-HS/Ctr group at each time point (*p*<0.01), the reduction was greater in the T-HS/EAA group (*p*<0.01).

### 3. Serum Hormones (Insulin and Corticosterone) Concentrations

**Figure 6:**
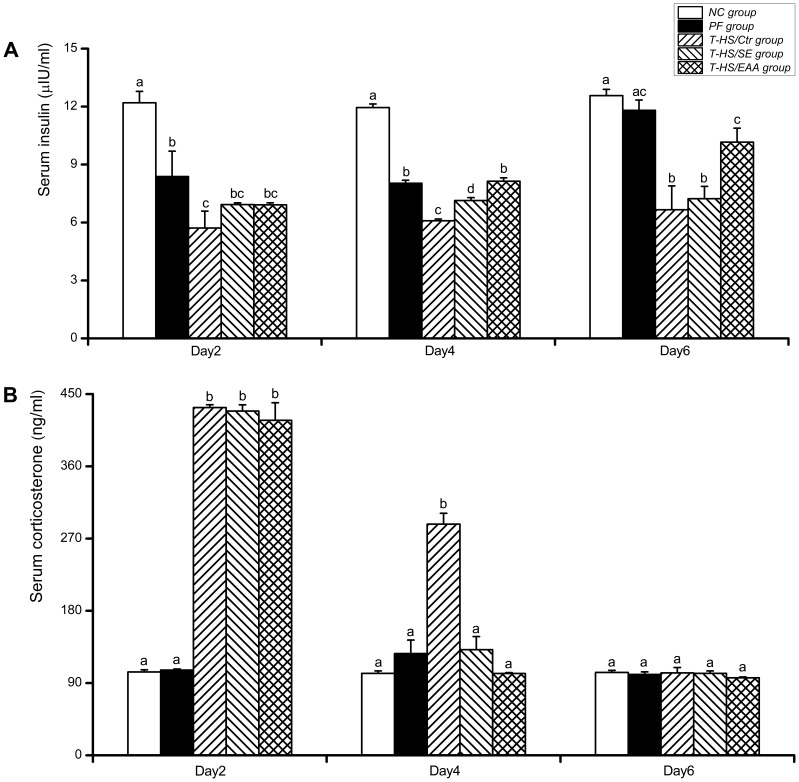
ELISA analysis of serum hormones concentrations. Serum concentrations of insulin (A) and corticosterone (B) in each group were measured following 2, 4, and 6 days of recovery from T-HS. Values are presented as mean ± SE. Groups with different letters at each time point indicate a significant difference (*p*<0.05). T-HS, trauma-hemorrhagic shock.

Insulin levels in the T-HS/Ctr group were significantly decreased compared with the PF group at all the time points (*p*<0.05, at day 2; *p*<0.01, at day 4 or 6; **Figure 6A**). Both enterally fed groups showed increase in insulin concentrations, but it failed to reach statistical difference compared with the T-HS/Ctr group at day 2. By day 4, standard and EAA enriched high-protein EN significantly increased insulin levels 1- and 1.3-fold, respectively, compared with the values detected in the T-HS/Ctr rats (*p*<0.01). Moreover, a greater magnitude of feeding-induced hyperinsulinemia was observed in the T-HS/EAA group than T-HS/SE group on days 4 and 6, respectively (*p*<0.01, *p*<0.05). Regression analysis of insulin and IGF-1 further indicated a significantly positive liner relationship (*y* = 0.038*x*−1.08; *R^2^* = 0.483; *p*<0.01).

Serum corticosterone concentrations in rats subjected to T-HS significantly increased by day 2 (*p*<0.01, **Figure 6B**). Concentrations in the T-HS/SE and T-HS/EAA groups gradually returned to normal as early as at day 4, whereas corticosterone levels in the T-HS/Ctr group remained statistically elevated (*p*<0.01). Meanwhile, corticosterone levels in the T-HS/EAA group appeared lower than T-HS/SE group on days 4 and 6, but without statistical difference.

### 4. Cell Signaling

**Figure 7:**
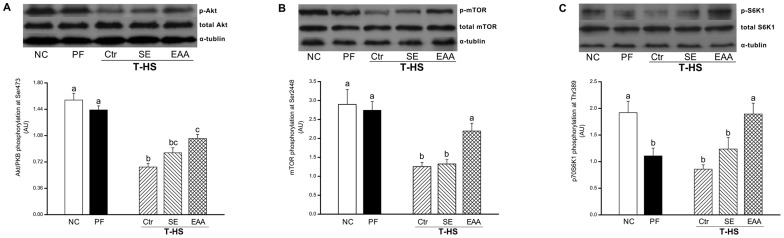
Phosphorylations of mTOR related signaling effectors in gastrocnemius at day 6. (A) Akt phosphorylation at Ser473 and total Akt. (B) mTOR phosphorylation at Ser2448 and total mTOR. (C) p70S6K1 phosphorylation at Thr389 and total p70S6K1. The density of each western blot is quantified by Quantity One software and the data are presented as mean ± SE in arbitrary unit (AU) from three independent experiments. Groups with different letters indicate a significant difference (*p*<0.05).

To further assess the signaling transduction associated with muscle protein synthesis, phosphorylations of several key proteins involved in mTOR signaling pathway were determined in gastrocnemius at day 6. After T-HS, phosphorylation of Akt at Ser473 in the T-HS/Ctr group was greatly suppressed (vs NC, *p*<0.01; vs PF, *p*<0.01; **Figure 7A**). Phospho-Akt levels in the T-HS/EAA group, rather than T-HS/SE group, were significant higher than the T-HS/Ctr group (*p*<0.01), coinciding with the fact that free IGF-1 levels peaked at day 6. Meanwhile, the phosphorylation of mTOR at Ser2448 in the T-HS/Ctr group decreased to 43.3% and 45.7% compared with the NC and PF groups, respectively (*p*<0.01, **Figure 7B**). A minor increase but without significance in phospho-mTOR levels was observed in rats receiving standard EN formula. However, in the T-HS/EAA group, mTOR phosphorylation at Ser2448 was significantly elevated (176%, vs T-HS/Ctr, *p*<0.05; 166%, vs T-HS/SE, *p*<0.05). As the important downstream effector of mTOR signaling, Thr389-phosphorylation of S6K1 was also measured. S6K1 phosphorylations in the PF and T-HS/Ctr groups were similar, but markedly lower than the NC group (*p*<0.05, *p*<0.01; **Figure 7C**). After EN treatment, S6K1 phosphorylation increased in both groups, but only with statistical significance observed in the T-HS/EAA group compared with the T-HS/Ctr group (*p*<0.01). Furthermore, phospho-S6K1 levels in the T-HS/EAA group were also higher than the T-HS/SE group (*p*<0.05).

### 5. Blood EAA Concentrations

**Figure 8:**
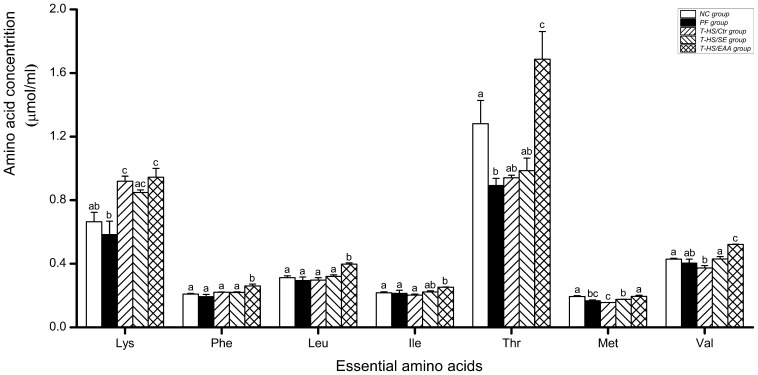
Blood essential amino acid concentrations at day 6. Values are presented as mean ± SE. Groups with different letters at each time point indicate a significant difference (*p*<0.05).

There was no significant decrease in branched chain amino acid (BCAA) concentrations in the T-HS/Ctr group compared with the NC or PF group at day 6, with the exception of valine (vs NC, *p*<0.05; **Figure 8**). Treatment with standard EN slightly, without significance, increased the blood leucine and isoleucine levels. In contrast, a pronounced increase in BCAA levels was detected in the T-HS/EAA group (vs T-HS/Ctr, *p*<0.01), and the leucine and valine concentrations were even higher than that observed in the T-HS/SE group (*p*<0.01). Similarly, blood levels of other EAA were increased after EN treatment, but with greater in the T-HS/EAA group (vs T-HS/SE, *p*<0.05).

## Discussion

Sustained suppression of circulating IGF-1 levels is one important metabolic derangement during critical illness, which is implicated in the pathogenesis of impaired muscle protein anabolism. Our study illustrates that EAA enriched high-protein EN favors early recovery of the IGF-1 system in T-HS injured rats, accompanying with the obvious muscle mass maintenance and downstream mTOR pathway activation, which indicates effective stimulation of muscle protein translation initiation. These results provide valuable insight into the metabolic regulation stimulated by high-protein nutrition support during the stress response to critical illness.

The composition of EAA supplementation used in this study contained a large proportion of leucine. Recently, leucine is shown to be capable of stimulating insulin secretion [23]–[25]. This is consistent with our data, which the insulin levels were highest in the T-HS/EAA group by day 4 compared with the T-HS/SE and T-HS/Ctr groups. As reported, insulin is a known regulator to inhibit hepatic IGFBP-1 production [26], and elevated IGFBP-1 levels under catabolic stress can act to inhibit the anabolic effect of IGF-1 [27]. In the present study, serum IGFBP-1 concentrations appeared to correlate well with these data, displaying dramatically decreasing levels that were consistent with the increase in circulating insulin levels observed in enterally fed rats at the time point coinciding with the emergence of elevation in serum free IGF-1 levels. And this response appeared more pronounced in the T-HS/EAA group than T-HS/SE group. Regression analysis of insulin and IGF-1 further indicated a strong positive correlation (*y* = 0.038*x*−1.08; *R^2^* = 0.483; *p*<0.01), which was in line with the previous work [18].

However, Ma *et al.* [28] found that hepatic IGFBP-1 gene expression was dramatically increased immediately following T-HS along with hyperinsulinemia at 90 and 210 minutes. This does not entirely fit with our results, which appears to depend upon the different time points selected. During the “ebb” phase of injury, insulin levels are commonly decreased or remain unchanged [29], [30]. Insulin requires the PI3K pathway for inhibition of hepatic IGFBP-1 production [31]. Hepatic insulin resistance, characterized by a defect in insulin-induced PI3K-Akt signal transduction, rapidly develops within 90 minutes following T-HS [28], but may have been alleviated within 48 hours post-T-HS in the present study.

Endogenous glucocorticoids also mediate IGFBP-1 production. Previous work demonstrated that pretreating animals with glucocorticoid receptor antagonist RU486 partially prevented burn-induced increase in IGFBP-1 and further contributed to the increase in plasma IGF-1 levels [32]. The enterally fed rats in the present study appeared to have an early reduction in serum corticosterone levels following T-HS, which is possibly related to the modulation of host defense by enteral nutrients [33]. This change was correlated with decreased IGFBP-1 as well as increased IGF-1 levels following 3 days of nutrition support.

Previous studies using a similar EAA formula or leucine alone to investigate the stimulatory effect upon skeletal muscle protein synthesis did not demonstrate changes in serum IGF-1 [20], [34], [35]. Despite various experimental conditions, those studies measured total IGF-1, instead of free levels. Meanwhile, acute dosing of EAA or leucine solution on the background of inadequate nutrient availability is not sufficient for stimulation of IGF-1 production. In our study, we provided a high-protein diet to the T-HS/EAA animals, with additional EAA supplementation based on the standard EN formula, to investigate the changes in free IGF-1 levels, which represent the “bioavailable” portion of the IGF-1 pool in the circulation [16]. Our study indicated that feeding with EAA enriched high-protein EN greatly increased serum free IGF-1 levels compared with the standard EN-fed rats as early as at day 4. This is a desirable outcome, since it has been concluded that IGF-1 plays an important role in skeletal muscle mass maintenance [36]. In our study, significantly increased muscle mass, especially in the fast-twitch gastrocnemius muscle, was identified 2 days following T-HS in the T-HS/EAA group.

We also detected that EAA enriched high-protein EN significantly enhanced the activation of mTOR signaling pathway; phosphorylations of mTOR and its downstream effector S6K1 in rats receiving EAA enriched high-protein formula increased nearly two-fold compared with those receiving standard EN. Recently, a large body of evidence has established that amino acid, especially leucine, can be considered as a signaling molecule to stimulate protein translation initiation through an Akt-independent pathway. Acute leucine ingestion can effectively increase protein synthesis and promote the mTOR-related signaling transduction in both normal and stress conditions [37]–[39]. The stimulation of muscle protein synthesis, as previously reported by Bohé *et al.* [40], was positively associated with blood EAA concentrations in a dose-response manner. In our study, blood amino acid analysis revealed that leucine and other EAA were significantly increased after EAA enriched high-protein EN treatment. Thus, we speculate that the response to muscle protein translation initiation induced by EAA enriched high-protein EN may be partly attributed to the nutrient-mediated mTOR signaling regulation.

The PI3K-Akt pathway, upstream of mTOR, can be phosphorylated by ligands including IGF-1 and insulin and that in turn stimulates the mTOR phosphorylation to induce the muscle protein translation initiation. Previous studies demonstrated the rapid development of insulin resistance in skeletal muscle following T-HS, with the earliest insulin signaling defect occurring at 60 minutes [41], [42]. Zhai *et al.* [43] further indicated that the T-HS-induced acute muscle insulin resistance was specific to 6- and 10- week old rats, but not the post-weaning. However, the insulin signaling defect is not permanent. Thompson *et al.* [44] reported that insulin-induced phospho-Akt signaling in skeletal muscle would be gradually restored by 5 and 24 hours following fluid resuscitation. Therefore, it can reasonably be inferred that muscle insulin resistance is a metabolic feature in the acute phase of T-HS, and the diminished signaling transduction associated with the PI3K-Akt pathway may be partially responsible for the increased muscle protein wasting following T-HS.

In our study, three later time points following T-HS were selected. Activated Akt was observed at day 6 in both enterally fed groups, but with greater in rats receiving EAA enriched high-protein EN, coinciding with the emergence of significant elevation in both insulin and IGF-1 levels. Previous studies demonstrated the protein retention effect of insulin was likely to be exerted by reducing the protein catabolism after trauma or surgery, because no correlation between changes in muscle protein synthesis and insulin sensitivity was found [45], [46]. However, administration of IGF-1 effectively attenuates the inhibition of protein synthesis in sepsis or trauma and further ameliorates the loss of muscle mass [47], [48]. Therefore, in addition to the nutrient-dependent mechanism described above, the increased mTOR anabolic signaling in the T-HS/EAA group may be partially due to IGF-1 signaling pathway activation.

A limitation to the current study is that it is not yet clear whether the increased serum free IGF-1 levels following EAA-enriched high-protein EN are due to the overall increase in IGF-1 production or reduced IGF-1 degradation. As mentioned previously, IGFBP-3 has been shown to be regulated by proteolysis. Increasing proteolytic cleavage of IGFBP-3 causes a decreased affinity for IGF-1, which is responsible for the decrease in circulating IGF-1 levels during stress [49]. In the present study, high serum free IGF-1 levels were observed in the T-HS/EAA group, which were quite consistent with increased IGFBP-3 concentrations. Therefore, further study is required to investigate the IGFBP-3 proteolysis activity and total IGF-1 levels following nutrition support, using a T-HS injured rat model, to make a better interpretation of our data [16].

In summary, our findings firstly demonstrate the effect of EAA enriched high-protein EN upon the metabolic regulation of skeletal muscle protein anabolism by regulating the IGF-1 system and downstream anabolic signaling transduction. We demonstrated that early nutrition support favored the recovery of serum free IGF-1 levels following T-HS, and the EAA enriched high-protein EN treatment appeared more effective. It was beneficial for the muscle mass maintenance in T-HS injured rats. Moreover, long-term treatment with EAA enriched high-protein EN effectively activated the mTOR related translation initiation factors, indicating the stimulation of muscle protein synthesis. And this response might be based on cooperation between IGF-1- and nutrient- mediated signal transduction pathways.
